# Supplementary figures and images for: Coupled exoskeleton assistance simplifies control and maintains metabolic benefits: A simulation study
Source: PLoS One. 2022 Jan 5;17(1):e0261318. doi: 10.1371/journal.pone.0261318 (PMC8730392; doi:10.1371/journal.pone.0261318)

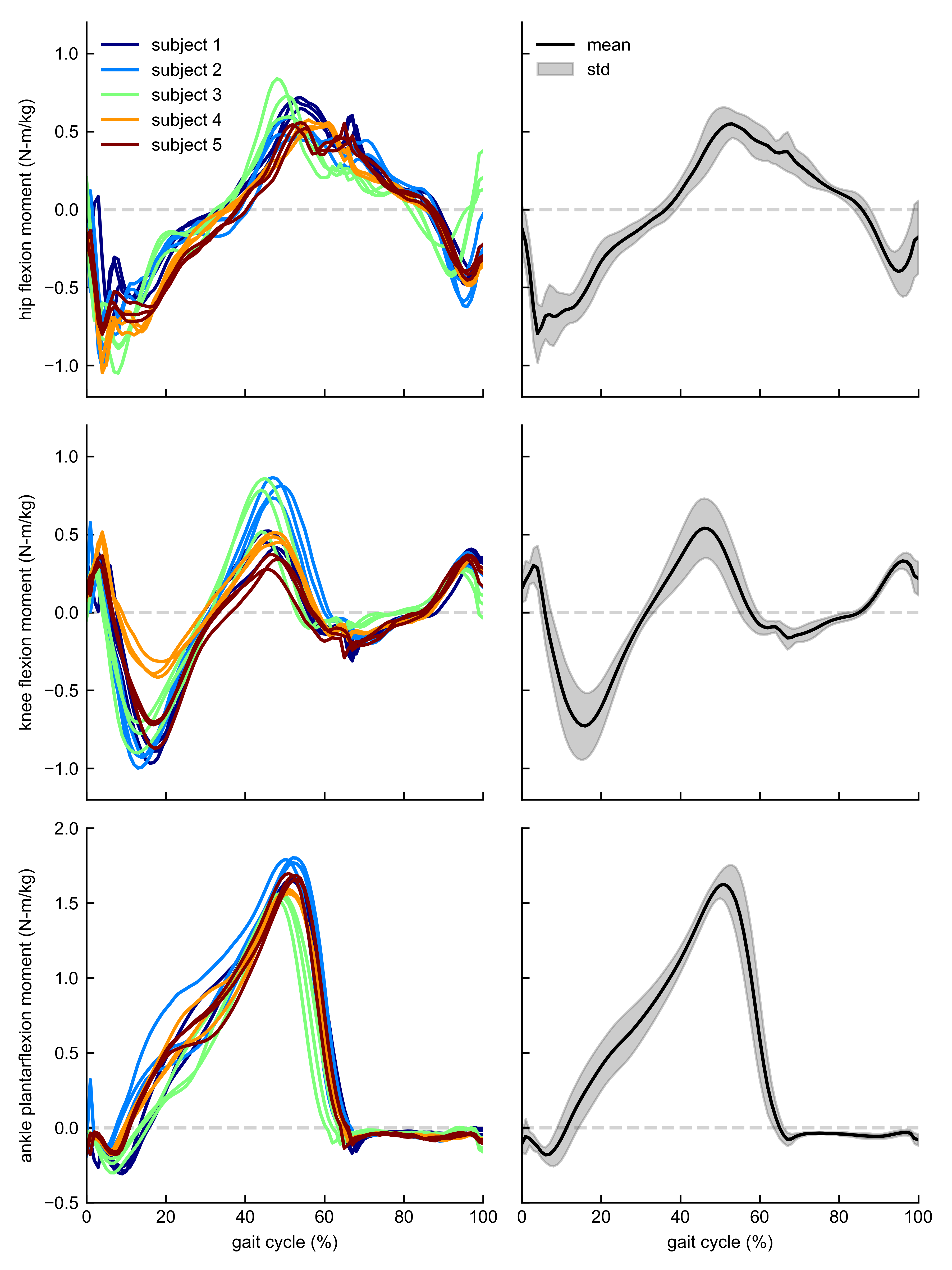

Supplement: S1 Fig — Left: net joint moments from inverse dynamics for individual subjects. Right: joint moment means (black) and standard deviations (gray bands) across subjects. (TIF) [file pone.0261318.s001.tif]

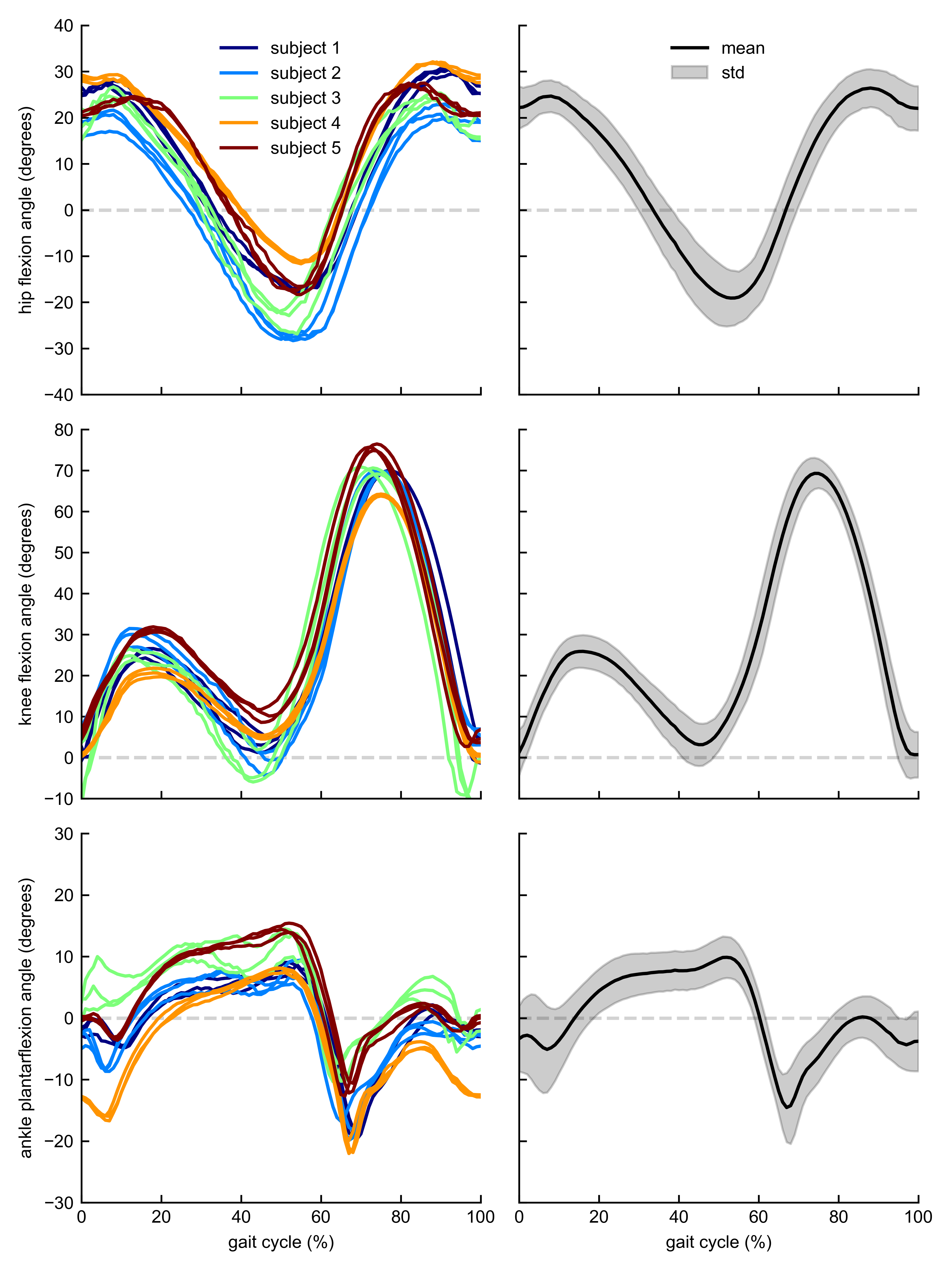

Supplement: S2 Fig — Left: joint angles from inverse kinematics for individual subjects. Right: joint angle means (black) and standard deviations (gray bands) across subjects. (TIF) [file pone.0261318.s002.tif]

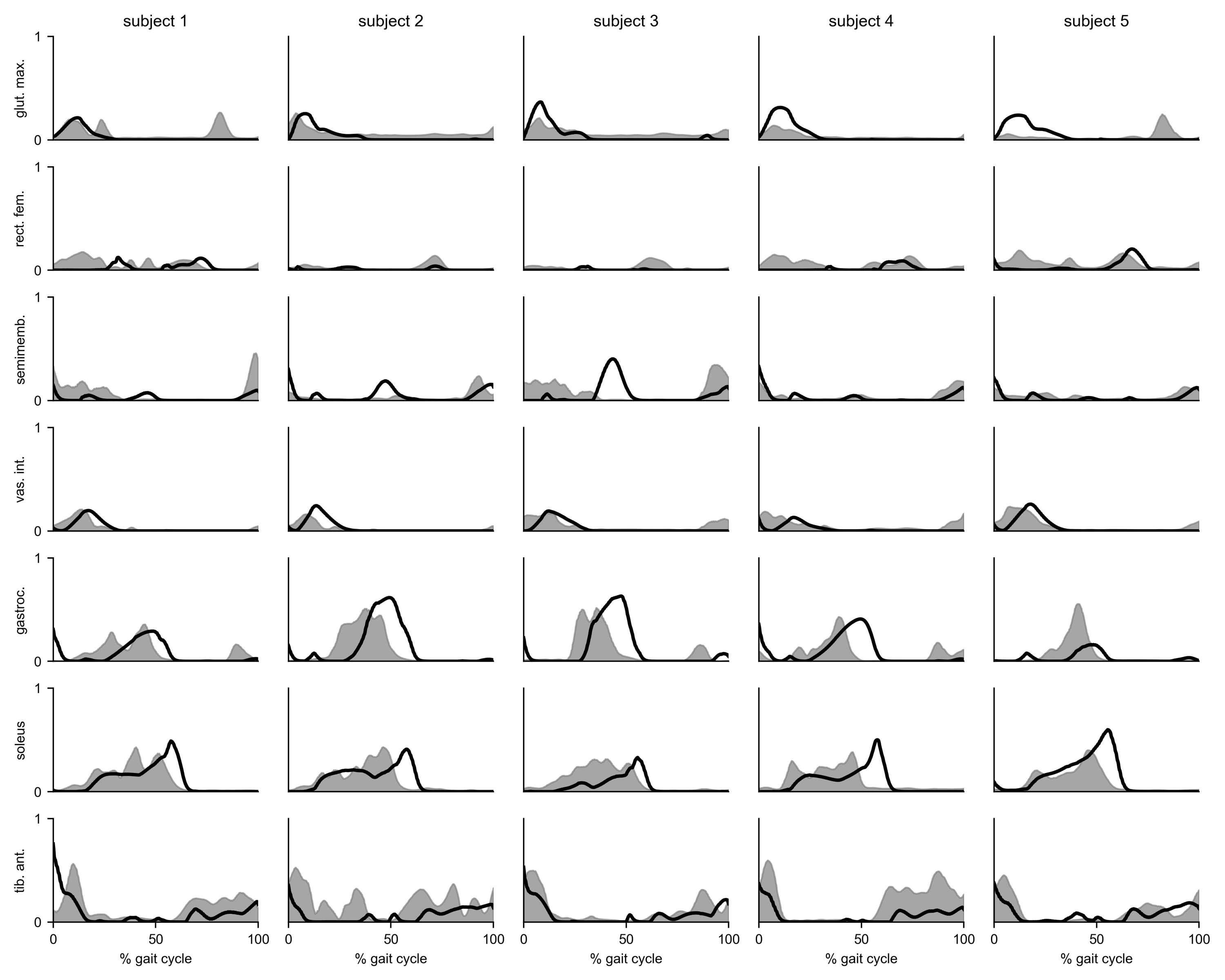

Supplement: S3 Fig — This figure shows electromyography data (gray bands) recorded from walking experiments compared to optimized activations generated from unassisted simulations (black). Both electromyography data and simulated activations are averaged across gait cycles not included in the muscle parameter calibration procedure. (TIF) [file pone.0261318.s003.tif]

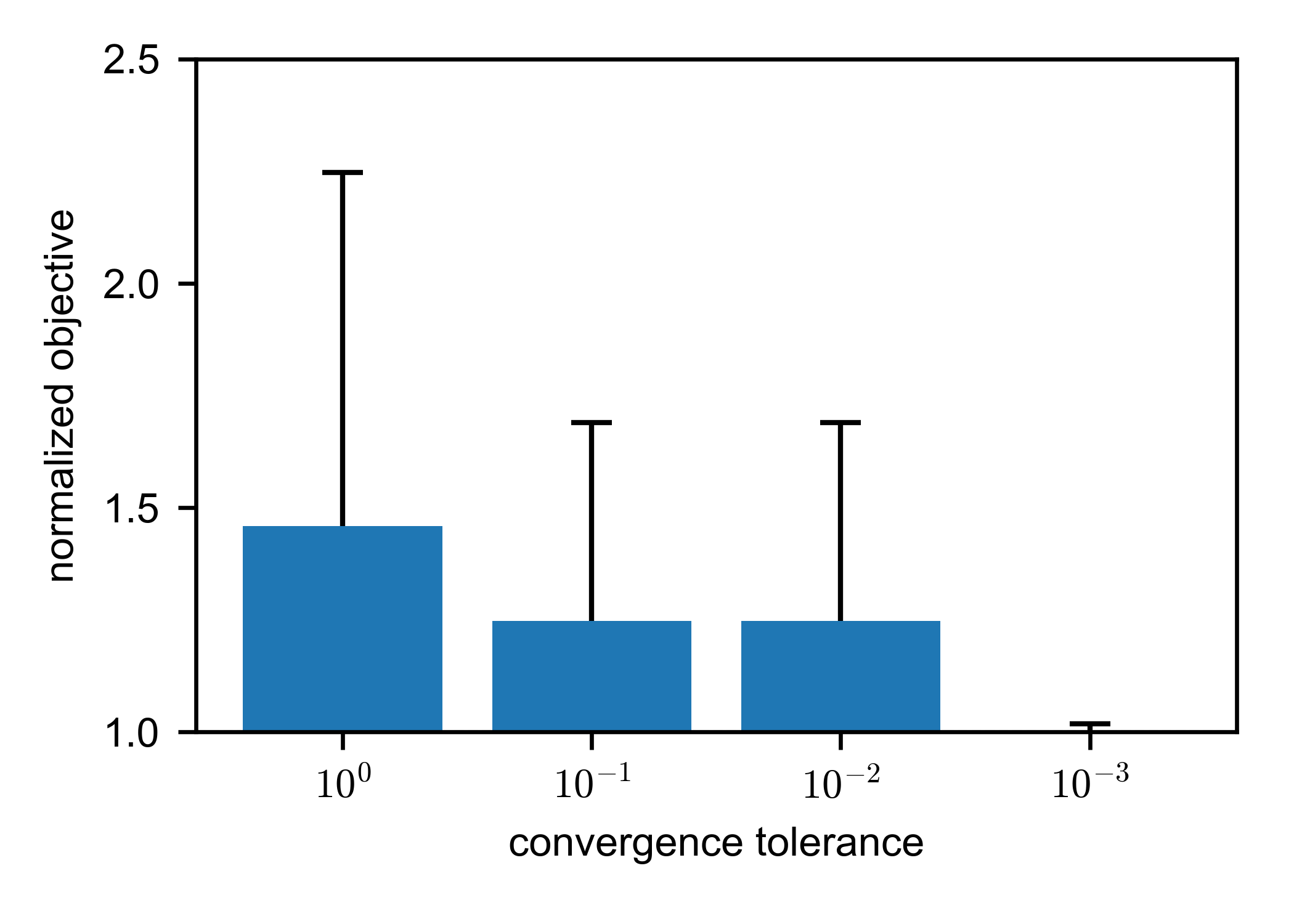

Supplement: S4 Fig — The mean (bars) and standard deviation (whiskers) of normalized objective values for unassisted walking solutions across subjects and gait cycles. Objective values at each convergence tolerance are normalized by objective values using a convergence tolerance of 10−4. We used a convergence tolerance of 10−3 to generate our results, since tightening the tolerance to 10−4 had little effect on the objective (i.e., the normalized objective values were close to one for the 10−3 tolerance). (TIF) [file pone.0261318.s004.tif]

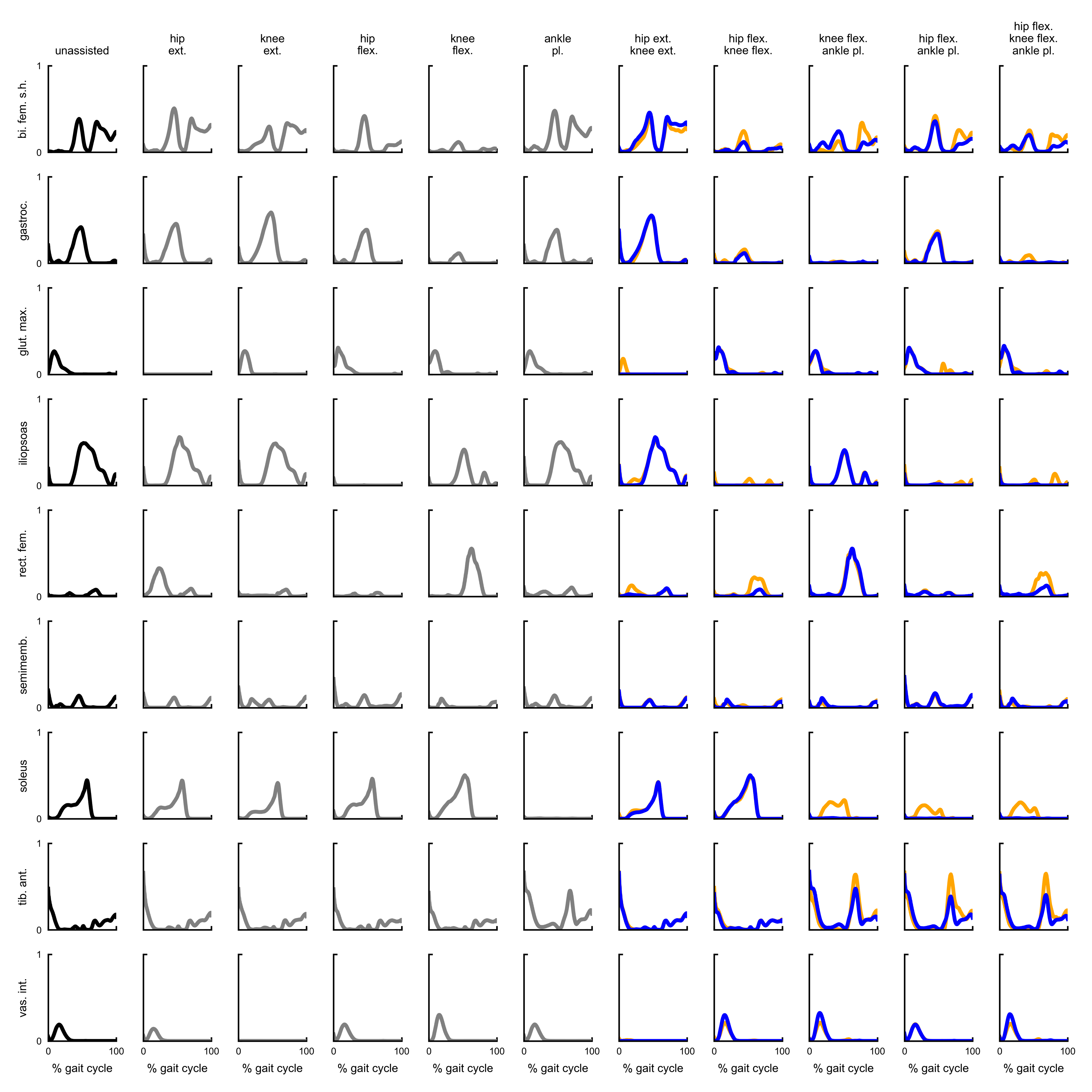

Supplement: S5 Fig — This figure shows muscle activations averaged across subjects for unassisted walking (black), single-joint assisted walking (gray), and multi-joint coupled (orange) and independent (blue) assisted walking. (TIF) [file pone.0261318.s005.tif]

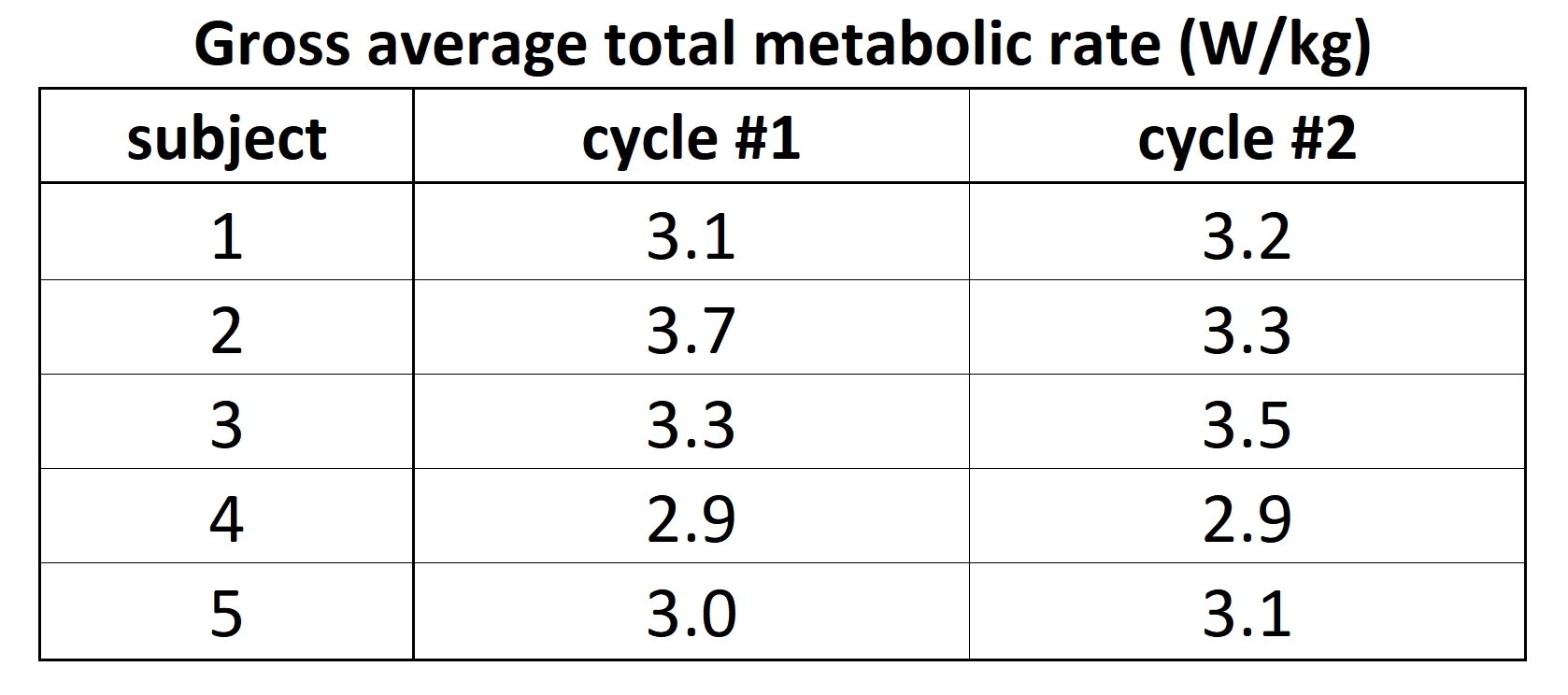

Supplement: S1 Table — This table shows the predicted gross average total metabolic rates for each subject. The columns represent the gait cycles used when testing single and multi-joint devices. These values underestimate experimental values typical of normal unassisted walking (4.0–4.3 W/kg, [48]). (TIF) [file pone.0261318.s006.tif]

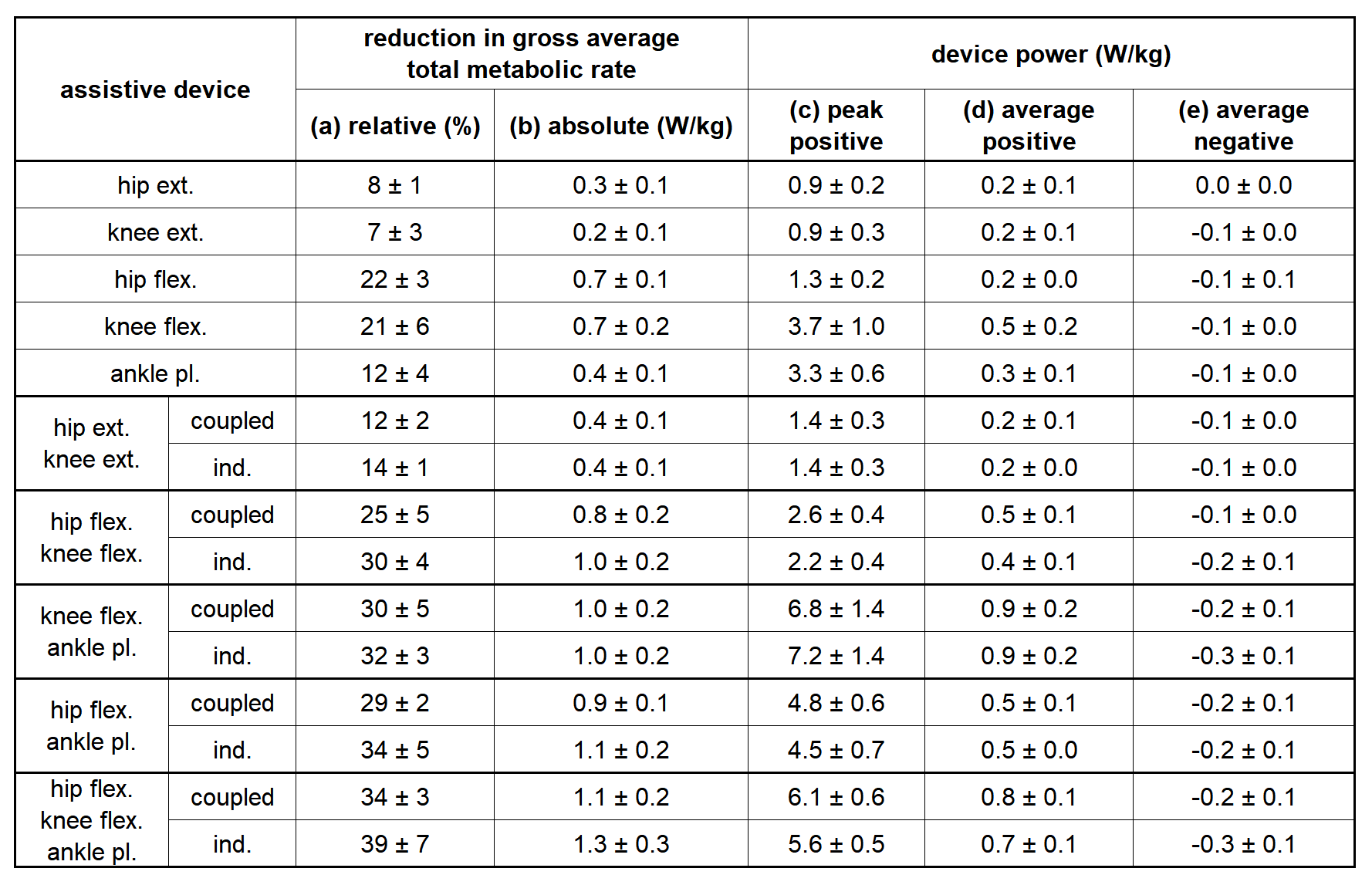

Supplement: S2 Table — This table shows (a) relative and (b) absolute reductions in gross average total metabolic rate and the (c) peak positive, (d) average positive, and (e) average negative power for each single and multi-joint device. Quantities in columns (b)-(e) are normalized by subject mass. All columns are reported as mean ± standard deviation across 5 subjects. (TIF) [file pone.0261318.s007.tif]

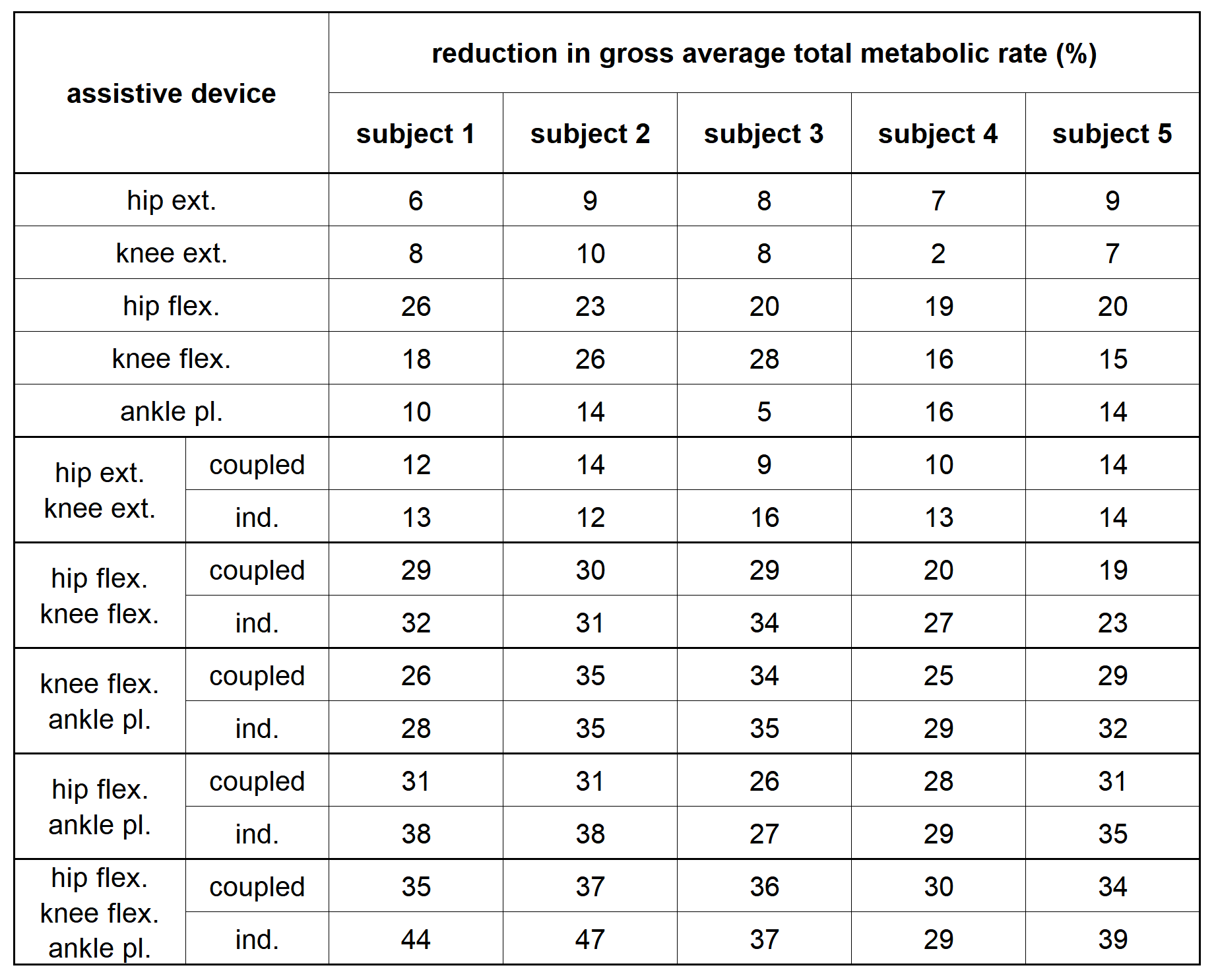

Supplement: S3 Table — This table shows subject-specific relative reductions in gross average total metabolic rate for each single and multi-joint device. All quantities are percent reductions in metabolic cost relative to unassisted walking. (TIF) [file pone.0261318.s008.tif]

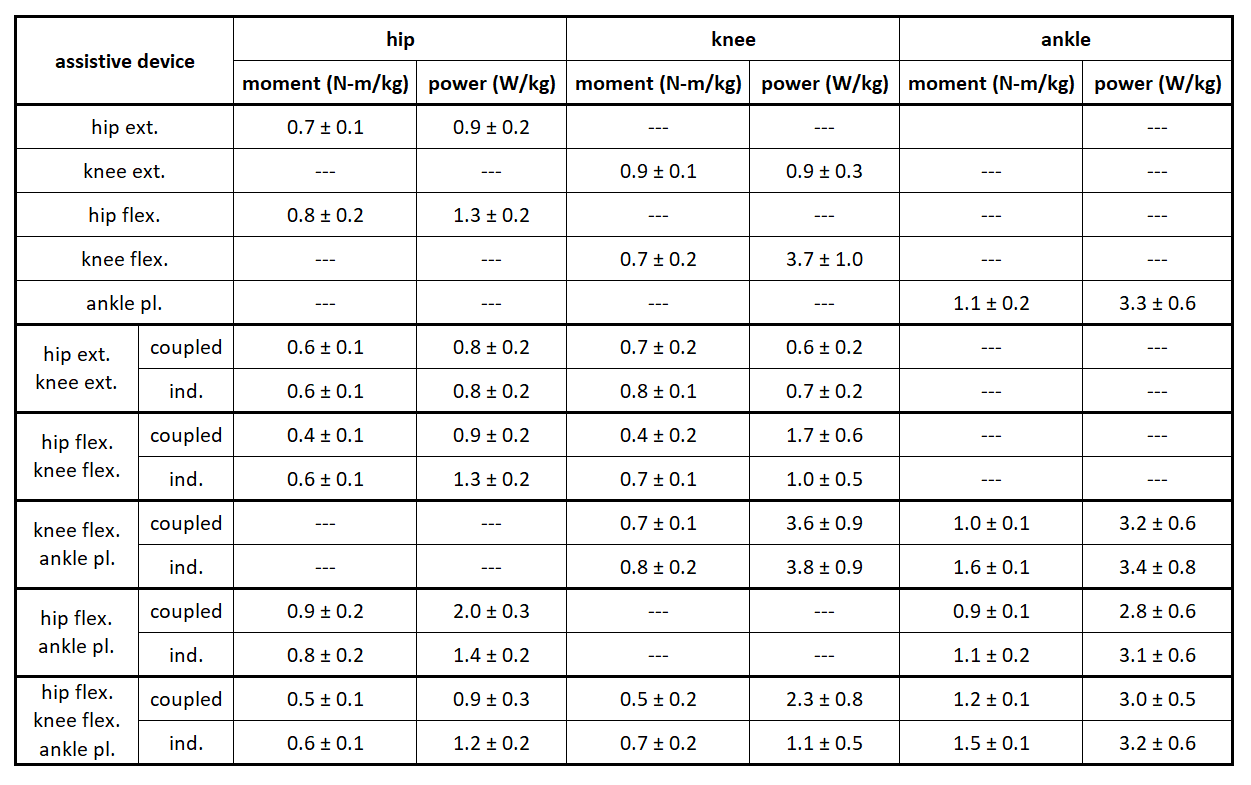

Supplement: S4 Table — This table shows the peak device moments and powers for individual degrees-of-freedom for each single and multi-joint device. All quantities are normalized by subject mass and are reported as mean ± standard deviation across 5 subjects. Peak moment values are peak magnitudes of device moments applied at each degree-of-freedom. (TIF) [file pone.0261318.s009.tif]
