# Supplementary material for: Coupled exoskeleton assistance simplifies control and maintains metabolic benefits: A simulation study
Source: PLoS One. 2022 Jan 5;17(1):e0261318. doi: 10.1371/journal.pone.0261318 (PMC8730392; doi:10.1371/journal.pone.0261318)
Supplement: Attachment — Submitted filename: RebuttalLetterRev2.pdf [file pone.0261318.s011.pdf]

(The editor's and reviewers' text is formatted with blue italics. Line numbers refer to the version of the revised manuscript without tracked changes.)

Reviewer #2

The authors have appropriately addressed my previous comments regarding the manuscript titled "Coupled exoskeleton assistance simplifies control and maintains metabolic benefits: a simulation study". While I am satisfied with the edits, there are some minor points that I think could be expanded upon which would increase the overall quality of the publication. These points are described below.

We are glad that our edits have addressed the reviewer's major concerns. We have further revised the manuscript to address the reviewer's remaining comments related to statistical testing and the value of this study in light of the limitations, as described in detail below.

Methods

After performing the requested statistical testing, there are several comparisons in which the null hypothesis could not be rejected. It would be worthwhile for the authors to speculate why these specific instances did not provide metabolic savings compared to the other coupled assistance systems. Could insufficient sampling be excluded as a possibility? Or is 5 participants and only 3 steps of locomotion imply insufficient to detect the metabolic savings? If the sampling is appropriate, why do only some of the coupled systems show metabolic savings?

We agree that insufficient sampling could explain why the null hypothesis could not be rejected for these devices. Accordingly, we have added a sentence on lines 368-371:

> Finally, we created simulations using experimental gait data from only five subjects, which may partially explain why some of the multi-joint devices we tested did not produce significantly different metabolic cost changes compared to single-joint devices.

We chose not to speculate broadly on why certain multi-joint devices did not produce significantly different metabolic cost changes compared to single-joint devices.

Discussion

The authors have, very appropriately, acknowledged several limitations, e.g. massless actuators, of their simulation study and have emphasized the necessity of experimental data to validate their findings. It may be worthwhile for the authors to expand on why they think these findings will be validated or why the assumptions they made are reasonable.

We are glad that the previous revisions adequately address the reviewer's comments about the limitations of our study. As suggested, we updated the discussion to note why our findings are

valid and assumptions are reasonable. These updates are included in the revised manuscript (line 372-388).

Minor Issues

*It would be very helpful to the readers to indicate on the figures which comparisons were statistically significant (with * or some other visual).*

As suggested, we have updated Figure 1 to include asterisks to visualize which multi-joint devices produced significantly different metabolic cost changes compared to their respective single-joint devices. We have also updated the Figure 1 caption to reflect the change.
